# Association of Physical Activity and Sleep Quality with Academic Performance Among Fourth-year MBBS Students of Rawalpindi Medical University

**DOI:** 10.7759/cureus.5086

**Published:** 2019-07-06

**Authors:** Muhammad Zubair Satti, Tayyab Mumtaz Khan, Qurat-ul-ain Qurat-ul-ain, Muhammad Junaid Azhar, Hassan Javed, Mariam Yaseen, Maria Taskeen Raja, Areeba Zamir, Muhammad Hamza

**Affiliations:** 1 Medicine, Rawalpindi Medical University and Allied Hospitals, Rawalpindi, PAK; 2 Psychiatry, Rawalpindi Medical University, Rawalpindi, PAK; 3 Psychology, Rawalpindi Medical University, Rawalpindi, PAK; 4 Psychiatry, Rawalpindi Medical University and Allied Hospitals, Rawalpindi, PAK

**Keywords:** pittsburgh sleep quality index, gpaq, mbbs

## Abstract

Background

Medical students face greater academic stress and devote more time to their studies due to the tough nature of medical education, at the cost of sleep and physical activity. Good sleep quality and physical activity improve the mental ability and academic performance of the students.

Objectives and rationale

The study aims to assess sleep quality and physical activity levels among fourth-year MBBS students of Rawalpindi Medical University. We compare these levels with gender and boarding status and correlate them with the academic performance of the students. This may provide new target areas to improve the academics of students performing below average.

Materials and methods

It was a descriptive, cross-sectional study conducted in March 2019 on 344 medical students enrolled in the fourth-year MBBS class of Rawalpindi Medical University. Sleep quality was assessed by the Pittsburgh Sleep Quality Index (PSQI), physical activity levels by the Global Physical Activity Questionnaire (GPAQ), and academic performance by the marks attained in the most recent pathology class test. The students who could not prepare for the test in the usual manner were excluded from the study. Two-hundred nineteen (219) students were part of the final study sample. Data analysis was performed using SPSS v.22.0 (IBM Corp, Armonk, NY, US). A chi-squared test, independent samples t-test, Pearson’s correlation, and a multiple linear regression model were used to assess the variables.

Results

Sleep quality and physical activity were significantly correlated with academic performance (p-values of the chi-square and t-test were <0.000). Pearson’s correlation coefficient was -0.69 for PSQI (p<0.000) and 0.62 for GPAQ (p 0.003) with test scores. Gender showed significant association with sleep and physical activity levels (male students had better physical activity level and poorer sleep quality than female students) but no association with test scores. Boarding status was significantly associated with all three variables. Boarders had lower mean test scores and poorer sleep and physical activity indices as compared to non-boarders. The multiple linear regression model was valid (p-value of the F test was <0.000), with beta coefficients of -2.53 ( p=0.002) for sleep quality and 1.37 (p=0.01) for physical activity. The R^2^ value was 0.84 (84%).

Conclusions

Our study indicates an overall poor sleep quality and physical activity level among fourth-year medical students, particularly boarding students, who have lower test scores and worse sleep and physical activity levels. In general, male students have better GPAQ scores and female students have better PSQI scores. Both the PSQI and GPAQ scores are significantly correlated with test scores and provide potential target areas to improve the exam performance of the students.

## Introduction

Medical students are future clinicians responsible for providing health care to others. They experience a relatively more competitive environment than students of other professions due to the tough and multidimensional nature of their course, learning to deal with patients and hospital staff and coping with the stress of acquiring clinical acumen alongside bookish knowledge [1-2]. Moreover, in developing countries, there are relatively fewer posts for doctors than the number of students enrolled in medical colleges [3]. The trend is not much different in the developed world as well, where one must strive to be on top in order to secure a good fellowship. Thus, almost every medical student has a constant pressure to perform better than his classmates in all routine class tests and exams for emerging successfully, with surety about their future as a clinician.

Since medical education is considered one of the toughest, medical students dedicate progressively more time to their studies at the cost of their physical activity and sleep duration, especially when tests or exams are near [4]. The proposed decrease in sleep and physical activities may also arise from the stressors mentioned above, which each medical student must face during the career.

Sleep quality significantly affects an individual’s daytime performance in any department [5]. Bad sleep quality adversely affects the memory, cognition, and attitude of the individual [6-7]. It is well-established that medical students have relatively poorer sleep quality [8]. We hypothesize that a student who does not sleep properly will also suffer regarding his academics because of the lack of concentration and decreased memory and learning abilities associated with improper sleep [9-10]. The implications of this assumption are manifold, as poorer academic performance will add to the student’s stress, further deteriorate sleep quality, and contribute to progressively worsening academic performances. The continuous stressors, along with poor sleep, may then eventually translate into adverse physical and psychological outcomes for the medical students [11]. Thus, it becomes important to routinely evaluate sleep quality levels in medical students.

Much research is focused on how physical activity effects mental and cognition abilities. It is well-established that a certain level of physical activity is needed for optimal mental function, and it also enhances concentration and helps cope with stress [12-14]. The dilemma is the fact that future clinicians themselves are reported to suffer from a poor index of physical activity [15]. Moreover, its effect on academic performance alongside sleep quality has not been evaluated in detail. We hypothesize that since a proper level of physical activity aids in coping with stress and enhances mental abilities, it may result in better academic performance for the students. Assessing the factors affecting the academics of medical students is important because academic performance accounts for the biggest stress of medical education: the stress to score well in all exams and class tests [16]. Students who cannot do so, despite trying their best, are predisposed to suffer from depression, which is particularly distressing in the medical profession.

The study aims to assess the levels of sleep quality and physical activity among the fourth-year medical students of Rawalpindi Medical University. We then compare these levels with gender and boarding status and correlate them with the academic performance of students. While gender-based differences in academics have been addressed in detail, we could not find studies that considered the difference between boarding and non-boarding students. The results of this study may provide a primordial level of intervention for medical students; promotion of healthy sleep habits and physical activity, especially for those students who are performing below average. Thus, the study may eventually identify two target areas for the management of medical schools to improve the academic performance of the students.

## Materials and methods

Study design

This descriptive, cross-sectional study was conducted in March 2019, during which 344 medical students enrolled in the fourth-year MBBS class of Rawalpindi Medical University were included in the study population. Two questionnaires were given to all students for a response rate of 74.71% (257 students returned properly filled questionnaires).

Assessment of the study variables

Assessment of Sleep Quality

The Pittsburgh Sleep Quality Index (PSQI) was used to assess the sleep quality of students over the past month. Its reliability to assess sleep quality has been established over the years [17]. Using a cut-off score of 5, PSQI has the following categories:

1 - A score of less than 5: It indicates good sleep quality.

2 - A score of 5 or greater: It indicates poor sleep quality.

Assessment of Physical Activity

The physical activity level was assessed by the Global Physical Activity Questionnaire (GPAQ) developed by the World Health Organization (WHO). GPAQ has been shown as a reliable instrument for measuring the level of physical activity [18]. Based on Metabolic Equivalent (MET) minutes per week, the following categories are made by GPAQ.

1 - High level of physical activity: It includes at least three days of vigorous physical activity, each day equaling at least 1500 MET-minutes per week and/or seven or more days of combined physical activity for at least 3000 MET-minutes per week.

2 - Moderate levels of physical activity: It includes at least three days of vigorous physical activity for at least 20 minutes per week and/or five or more days of moderate intensity and/or walking for at least 30 minutes per day and/or at least five days of a combination of physical activities and/or walking for at least 600 MET-minutes per week.

3 - Low levels of physical activity: It includes physical activity not meeting the above criteria.

Assessment of Academic Performance

Marks (out of 100) and percentile-based scoring of the most recent pathology class test, which happened three days before data collection was asked by a question added in PSQI to assess academic performance. Percentile-based scoring was used to ensure an easier comparison among students. The students who did not appear in that class test or who could not prepare for it in their usual manner due to illness, travel, or any unrelated event, were not included in the study. Two-hundred nineteen (219) students constituted our final sample based on these criteria. Based on their percentile, the students were divided into the following two groups:

1 - High achievers: This includes students in the 70th percentile or above.

2 - Low achievers: This includes students below the 70th percentile. 

Data analysis

A descriptive analysis of the study variables was performed using SPSS v.22.0 (IBM Corp, Armonk, NY, US). Cronbach's alpha of the questionnaires was calculated for 65 responses, to assess their reliability in our population. It was 0.81 for PSQI and 0.77 for GPAQ, indicating high interscale reliability. A chi-squared test and Pearson's correlation were used to see the direction and strength of the association between sleep quality, physical activity, and test scores. The independent samples t-test was used to see if study variables differ according to the gender and boarding status of the students. The predictive capacity of sleep quality and physical activity level for test scores was assessed by the multiple linear regression model. A p-value of less than 0.05 was considered statistically significant.

## Results

Population parameters and their cross-tabulations with study variables are provided in Table 1.

**Table 1:** Population parameters and their cross tabulation with study variables

Parameter		Cross-tabulation and chi-square analysis						
		Academic Performance		Physical Activity Level			Sleep Quality	
		Low	High	Low	Moderate	High	Poor	Good
Total count (219 students)		135 (61.6%)	84 (38.4%)	88 (40.2%)	95 (43.4%)	36 (16.4%)	132 (60.3%)	87 (39.7%)
Gender	Males (n=94)	60 (63.8%)	34 (36.2%)	26 (27.7%)	49 (52.1%)	19 (20.2%)	65 (69.1%)	29 (30.9%)
	Females (n=125)	75 (60.0%)	50 (40.0%)	62 (49.6%)	46 (36.8%)	17 (13.6%)	67 (53.6%)	58 (46.4%)
	p-values	0.56		0.005			0.001	
Boarding status	Boarders (n=117)	91 (77.8%)	26 (22,2%)	61 (52.1%)	41 (35%)	15 (12.8%)	85 (72.6%)	32 (27.3%)
	Non-boarders (n=102)	44 (43.1%)	58 (56.9%)	27 (26.5%)	54 (52.9%)	21 (20.6%)	47 (46.1%)	55 (53.9%)
	p-values	<0.000		0.001			< 0.000	
Academic performance and physical activity had a significant association (p<0.000)								
Academic performance and sleep quality had a significant association (p<0.000)								

Table 2 indicates the difference in PSQI and GPAQ scores between high and low achievers and shows the direction and strength of the association of these scores with students’ test scores by Pearson’s correlation. The correlation coefficients were statistically significant. It was negative for the PSQI score, meaning that a higher PSQI score correlates with lower test scores.

**Table 2:** Correlation of study variables with exam performance and test scores PSQI: Pittsburgh Sleep Quality Index; GPAQ: Global Physical Activity Questionnaire; MET: Metabolic Equivalent

Parameters	Exam performance			Pearson’s correlation	
	High achievers	Low achievers	Independent sample t-test (p-value)	Correlation coefficient (r)	p-value
PSQI score	7.5±2.1	12.9±3.7	<0.000	-0.69	<0.000
GPAQ score (MET-minutes per week)	805±75	631±49	<0.000	0.61	0.003

Table 3 summarizes the results of the independent samples t-test, showing that boarders had lower test scores, higher PSQI scores, and lower GPAQ scores than non-boarders. Gender-based differences are also shown in the table.

**Table 3:** Difference in study variables regarding gender and boarding status PSQI: Pittsburgh Sleep Quality Index; GPAQ: Global Physical Activity Questionnaire; MET: Metabolic Equivalent

Parameter	Gender			Boarding Status		
	Males	Females	Independent samples t-test (p-value)	Boarders	Non-boarders	Independent samples t-test (p-value)
Mean PSQI score	10.3±4,3	8.9±2.1	0.002	12.7±3.2	9.4±2.5	<0.000
Mean GPAQ score (MET-minutes per week)	769.5±63.7	651.3±57.9	0.01	635.8±61.1	759.5±71.3	0.003
Mean test score (out of 100)	78.1±4.7	82.3±6.2	0.67	74.9±4.1	84.5±6.7	<0.000

Table 4 shows the results of the multiple linear regression model, which was a valid model, as the F test was highly significant (p <0.000). The R^2^ value was highly significant at 0.84 (84%). The beta coefficient was negative for PSQI and positive for GPAQ, with significant p-values.

**Table 4:** Multiple linear regression model for test scores PSQI: Pittsburgh Sleep Quality Index; GPAQ: Global Physical Activity Questionnaire; MET: Metabolic Equivalent; CI: Confidence Interval

Variables	Unstandardized Regression Coefficient (B)	95% CI	p-value
PSQI score	-2.53	-1.29 to -4.05	0.002
GPAQ score (MET-minutes per week)	1.37	0.72 to 2.99	0.01
The Regression Model was significant (p-value of F test was <0.000)			
R^2^ value was 0.84 (84%)			

## Discussion

The results of our study provide valuable insights regarding the performance trends of medical students. Particularly important are the gender and residence-based differences in sleep and physical activity levels and their correlation with test scores.

We first observed the association between the sleep quality, physical activity, and exam performance of fourth-year medical students. A chi-squared analysis indicated highly significant p-values for both associations. In the next step, to find the direction and strength of the association between variables, Pearson’s product moment correlation was used. It showed a moderate to strong association between PSQI, GPAQ, and test scores. The correlation coefficient of PSQI was -0.69, showing that a higher PSQI score and hence poorer sleep quality is associated with lower test scores among the students. This lower exam performance due to poorer sleep quality is supported by studies conducted earlier, thus validating our results [11,19]. The GPAQ score showed a correlation coefficient of 0.61, meaning that higher GPAQ scores lead to better test scores. A study conducted in 2018 also identified a significant association between physical activity and academic performance, with self-esteem as an important mediator between the two [20]. Another study published in a sports medicine journal endorses our finding that physical activity leads to better academic outcomes [21].

Next, we applied these findings to our study population and observed whether the gender and boarding status-based distribution of medical students differ regarding the two concerned parameters and whether these parameters affect the test scores in the study. Descriptive statistics showed that male students had better physical activity and relatively poorer sleep quality as compared to female students. The higher physical activity levels of male students are explained by more opportunities to participate in outdoor sports whereas the majority of the female students do not have the facility due to cultural norms, particularly in the setting of a developing South-Asian country like Pakistan.

However, the relatively poorer sleep quality of male students is rather an outstanding finding because the females are generally believed to have a pre-disposition towards poorer sleep quality [22]. This finding has been reported regarding the general female population and studies regarding female medical students are scarce. An Austrian study conducted on medical students reported a similar finding of lower sleep quality in females, which contrasts with our study [23]. When the responses of PSQI questionnaires were investigated to find the cause of the higher PSQI scores of male students, we found that they had reduced sleep efficiency and greater sleep disturbances than their female counterparts. The reduced sleep efficiency of males was statistically significant (p =0.001), which means that male students tend to stay in bed longer than the hours of actual sleep. They tend to go to bed earlier and wake up late as compared to female students. This finding also contrasts with the general population trend where females are reported to have higher sleep latency [24]. Thus, the findings of our study indicate that females have different sleep patterns based on their strata, which should be investigated further.

The gender-based distribution showed no statistically significant difference between mean test scores. A plausible explanation is the fact that each group had one better index (females had better sleep quality whereas males had better physical activity levels), which may have canceled each other’s effects, leading to no statistically significant difference in the mean test scores. The gender-based difference in academic performance has conflicting literature, with some studies reporting no statistically significant difference between the genders and others indicating that female students outperform their male counterparts [25-26].

Further, when the results are looked at by dividing students according to their boarding status, the boarders are at a disadvantage regarding their sleep and physical activity habits. This factor has not been investigated by previous studies. The finding is quite understandable, as the boarders have no check and balance upon their routines. Moreover, the cult following of staying awake till late night and watching a lot of TV shows contributes to these declined indices. Also, these students, being far from their homes, feel a sense of loneliness, which decreases their sleep quality and motivation to participate in physical activities. These worsened scores on PSQI and GPAQ then translate into lower mean test scores for boarding students. The results of our study can be extrapolated to boarders of all types of professions because the same factors are in play in almost all hostels. Thus, two possible contributing factors regarding the lower performance of boarders are indicated by our study, which may provide means to improve the academic statistics of boarder students.

To statistically support the above findings and observe whether these indices can be used to predict exam performance, a multiple linear regression model was drawn using the PSQI and GPAQ scores as continuous predictors of the dependent variable test scores. Multiple linear regression was used to eliminate the confounding effect of two indices observed in separate simple regression analyses. Multicollinearity was excluded by a non-significant correlation between PSQI and GPAQ scores in Pearson's correlation. The model was significant (p-value of F test was <0.000) and explained 84% of the variations in the test scores of students. The equations of the model are:

y (outcome variable) = B × (predictor variable ) + Constant

Test score = -679.27 - 2.53 × (PSQI score) + 1.37 × (GPAQ score)

Here again, the beta coefficient of PSQI is negative, indicating that the rise in PSQI score predicts a decreasing test score.

Overall, we observed that 60% and 40% of all students have inappropriate sleep and physical activity levels, respectively, which is alarming and indicates immediate counseling and necessary interventions for the students, especially the boarders, to improve these figures. Poor overall sleep quality among university students is reported by another study as well [27].

The potential target areas for female students is to enhance their physical activity by providing them with institutional-based sports facilities and ensuring their proper participation. One method to make that possible is by including physical activities as part of the academic assessment of students. For males, the school management should aim at counseling regarding sleeping habits. Lastly, since the boarders suffered most regarding the indices and had lower test scores, the hostel management should implement policies regarding the routines of these students and attempt to improve their sleep and physical activity habits by disciplinary measures, such as a defined closing time of hostel gates and room lights and fining the students who do not obey the schedule. Moreover, the hostel management can organize sports competitions to enhance the physical activity levels of the boarders. These measures may improve the PSQI and GPAQ indices and lead to a non-academic intervention that improves the academics of the students.

## Conclusions

Our study indicates an overall poor sleep quality and physical activity level among fourth-year MBBS students of Rawalpindi Medical University, particularly the boarding students, who have lower test scores and worse sleep quality and physical activity indices. In general, male students have better GPAQ scores and female students have better PSQI scores. Both the PSQI and GPAQ scores are significantly correlated with test scores and provide potential target areas to improve the exam performance of the students. The management of medical schools needs to intervene and improve these statistics and conduct routine studies to assess the outcome of the interventions.
